# Supplementary material for: Prevalence of refractive error among Dongarias and use of Folding Phoropter (FoFo) as a field device enabling implementation research in this indigenous community. Tribal Odisha Eye Disease Study (TOES) Report # 13
Source: PLoS One. 2023 May 11;18(5):e0284790. doi: 10.1371/journal.pone.0284790 (PMC10174504; doi:10.1371/journal.pone.0284790)
Supplement: Attachment — Submitted filename: PONE-D-22-33033 review 12-29-2022.docx [file pone.0284790.s002.docx]

PONE-D-22-33033 Prevalence of Refractive error among Dongarias and Use of Folding Phoropter (FoFo) as a Field Device enabling Implementation Research in this Indigenous Community.Tribal Odisha Eye Disease Study (TOES) Report # 13

General comments:

This paper is based on a very interesting project carried out in a remote tribal area of India amongst a population with very special health care, demographic, and socio-economic characteristics that makes typical scientific study and reporting difficult or perhaps impossible. Thus, the methods used in this project are by necessity different than what typically gets studied and reported in the medical literature. This poses a number of difficulties that the authors do a great job in mitigating in order to provide important and very useful information to the audience. They were forced to do things in a manner that perhaps would otherwise not be acceptable in a more typical study, but in the context of the community they were working with, nonetheless valuable and most importantly, really interesting and creative. It also necessitates my reviewing this paper in a different manner than I typically do. I chose to do editing of the document that was submitted within the document itself and add additional comments where I believe indicated and useful to the authors. My edits are in both italics and a yellow colored font.

I am certain that this paper deserves publication in PLOS ONE with some additional editing by the authors. Given some allowances for the points above, this paper is different in some ways than a “typical” publishable research paper. However, it is perhaps particularly appropriate for a journal like PLOS ONE to give an opportunity to these authors that might be less acceptable to more “traditional” journals.

The authors do point out the many limitations created by the population and environment this study was conducted in. It might nonetheless benefit by some expansion of those limitations, as I suggest within my comments and edits within the paper. I have made an effort throughout the paper to make edits on language, syntax, and usage, which the authors may take or leave, but I think help make it a better and clearer paper.

Beyond the general comments above, a few more suggestions. First, you need a better description of the FoFo within this paper, and not just in references. A photo or diagram of the device, and maybe a photo of a subject undergoing the self-refraction process with the FoFo, would make clear to the reader what you are actually doing.

A better description of your definitions and categorizations of refractive error numerically in or adjacent to the refractive error table would be useful.

How are you defining literacy, or for that matter, non-literacy?

I was not clear how you calculated or used the term odds ratio in a later table. Perhaps define / clarify that better.

Finally, at the end of the paper, you come across a bit too speculatively for why the refractive errors are what this population has in comparison to other populations. Seems like a pretty far reach to me in some of your speculations. You might consider tempering things here.

This is a valuable contribution to the literature and with some mild revision, worthy of publication in PLOS ONE.
