# Supplementary material for: Prevalence of refractive error among Dongarias and use of Folding Phoropter (FoFo) as a field device enabling implementation research in this indigenous community. Tribal Odisha Eye Disease Study (TOES) Report # 13
Source: PLoS One. 2023 May 11;18(5):e0284790. doi: 10.1371/journal.pone.0284790 (PMC10174504; doi:10.1371/journal.pone.0284790)
Supplement: Attachment — Submitted filename: Point-to-point_Rebuttal.docx [file pone.0284790.s003.docx]

| **Reviewer’s Comment**  1.How was written consent obtained at the secondary centre?  2.Please clarify this: The prevalence of URE, the mean, median, and range were calculated using statistical analysis. What exactly was calculated for mean, median, and range is unclear.  3.Better description of FoFo:  First, you need a better description of the FoFo within this paper, and not just in references. A photo or diagram of the device, and maybe a photo of a subject undergoing the self-refraction process with the FoFo, would make clear to the reader what you are actually doing.  4.Description of definitions and categorizations of refractive error numerically:  5.Definition of Literacy:  6.Use of odds ratio:  7.Review of reference list:  8. Finally, at the end of the paper, you come across a bit too speculatively for why the refractive errors are what this population has in comparison to other populations. Seems like a pretty far reach to me in some of your speculations. You might consider tempering things here. | **Modifications**  1.This is added.  (Page no.7 and line 134-35).  2.Additional information are added.  (Page no.8 line 154-55)  3.The FoFo is described, advantage-disadvantage tabulated and appropriate images inserted. (line 114-18 &143-44 & 211-29 and Figure 1 and 2 & Table 6 and page 6-8 & 14-15).  4. It is added. (Page 7 and line 122-31).  5. It is now added. (Page 8 and line 150-51).  6. We have done it. (Page 8 and line 157-60)  7.One reference was added to the discussion's opening line (Page 18 and line 298-99)  The reference # 7 is corrected.  8. We thank the reviewer for the comment. We have deleted the following sentences - But we suppose, the higher prevalence of refractive error in females may possibly be due to their engagement in demanding near-tasks like shawl weaving. In general, a relatively higher prevalence of hypermetropia in females may be attributed to the reported shorter axial length, and decrease in anterior chamber depth and lens thickness in women than men. The higher prevalence of myopic astigmatism in males noted by us does not match other similar studies. We suspect it could be due to an increased involutional lid laxity; it was beyond the scope of the current study to measure | **Justifications (if no changes are made)**  -  -  -  -  -  - |
| --- | --- | --- |
